# A Review of 3,4-methylenedioxymethamphetamine (MDMA)-Assisted Psychotherapy

**DOI:** 10.3389/fpsyt.2019.00138

**Published:** 2019-03-20

**Authors:** Ben Sessa, Laurie Higbed, David Nutt

**Affiliations:** Neuropsychopharmacology Unit, Department of Medicine, Imperial College London, London, United Kingdom

**Keywords:** MDMA (3, 4-methylenedioxymethamphetamine), trauma, addiction, psychotherapy, alcohol

## Abstract

This paper provides a brief review of the history, proposed pharmacological mechanisms, safety issues, and clinical applications of the medicine 3,4-methylenedioxymethamphetamine (MDMA). Most clinical MDMA research in patients to date has focused on MDMA-assisted psychotherapy to treat posttraumatic stress disorder (PTSD). In this review paper other potential therapeutic applications for MDMA therapy are described, including contemporary studies treating anxiety associated with autism and the authors' ongoing study exploring the potential role for MDMA-assisted psychotherapy to treat alcohol use disorder. MDMA therapy for PTSD is now entering the final Phase 3 stage of drug development, with a target set for licensing by the FDA and EMA in 2021. This means that if clinical efficacy criteria are achieved, MDMA would become a medicine.

## The Early Therapeutic Use of MDMA

In the late 1960's, after lysergic acid diethylamide (LSD) was banned, some psychedelic therapists began exploring other drugs as tools to enhance psychotherapy. One, Leo Zeff, was initially introduced to MDMA in 1976 by psychedelic chemist, Alexander “Sasha” Shulgin, who had been studying psychedelics since the early 1960s (1). Zeff went on to successfully and safely give MDMA, then legal, to many thousands of patients (2). Shulgin, alongside chemist David E. Nichols, published the first report into the effects and pharmacology of MDMA in humans (3).

Not a “classic” psychedelic drug, but an “entactogen” (4), MDMA produces a more gentle and easily tolerated state compared to LSD. It is shorter-acting, which makes it more clinically manageable, it enhances feelings of empathy and bonding and allows users to access and process memories of emotional trauma (5).

Psychotherapists using MDMA in the early 1980s, when was called “Adam” or “Empathy,” wished to keep it within the clinical research community. But MDMA became rebranded as the more marketable “Ecstasy” and its non-clinical use spread—especially in the club scene or in large parties called raves. In 1984, in response to rising police seizures of the drug, the DEA announced that it intended to ban the compound. The clinical MDMA research community requested a hearing to debate the DEA's intention, but in May 1985 MDMA was initially placed in an emergency Schedule One category and subsequently became permanently scheduled thereafter, where it has stayed ever since—hugely restricting opportunities for its research (6). Due to this *Catch 22* situation, very little clinical research was able to take place. This prompted the formation of the US-based research organization, *The Multidisciplinary Association for Psychedelic Studies* (MAPS), which today is spearheading global clinical research of MDMA.

In the mid-eighties, a series of uncontrolled case studies, conducted before the ban, were published. These described the effective use of MDMA with individuals, couples and groups (7, 8). In 1988 the *Swiss Medical Society for Psycholytic Therapy* conducted individual and group psychotherapy with MDMA and LSD. Over a 100 patients with a wide range of psychiatric problems received an average of eight therapeutic sessions. Over 90% of patients described improvements at 19-months follow-up (9). But in 1993 the Swiss Ministry of Health withdrew permission to continue prescribing MDMA and LSD from the Swiss psychiatrists in the wake of concerns about the lack of research methodology and secondary to an ibogaine-related death of a patient (10). The compassionate use of MDMA has restarted in Switzerland in the last years and currently a few patients are treated each year based on individual authorizations by the Federal Office of Public Health.

Throughout the 1990s, tensions developed between the clinical MDMA community, who proposed MDMA was safe in controlled circumstances, and the media and politicians who favored strict prohibition to control recreational use. During this decade the UK brewing industry sponsored widely publicized anti-Ecstasy campaigns in response to their business being eroded by Ecstasy use (11). Undeterred by the political challenges, MDMA clinical research continued, with a MAPS-sponsored clinical study gaining approval in 2000 to look at MDMA for PTSD in Spain. But after just 1 year, a political backlash by the Spanish government shut down the study.

## Contemporary Clinical Research With MDMA

The first controlled clinical study demonstrating MDMA-assisted psychotherapy was eventually published in 2010—with impressive results (12). Twenty patients with treatment-resistant PTSD received, during a course of non-drug psychotherapy, either inactive placebo or two or three sessions of MDMA (initial dose of 125 mg, followed 2 h later by a further booster of 62.5 mg). At two and 12-month follow-up, 83% of the experimental group no longer met the criteria for PTSD, compared with just 25% of the patients in the placebo group. There were no drug-related serious adverse events and no adverse neurocognitive effects (12). Long-term follow-up of the cohort of successfully-treated patients demonstrated that remission from PTSD was maintained for up to 6 years (17 to 74 months, mean of 45 months), without having any further doses of MDMA (13).

A second, smaller MAPS-sponsored study in 2013 again explored the potential for MDMA Psychotherapy for treatment-resistant PTSD and showed substantial improvements (14). This study by Oehen was smaller than Mithoefer's and although there was a definite trend in the direction of MDMA therapy being superior to placebo, at first sight the statistics failed to demonstrate a significant reduction in CAPS for the experimental subjects (14). However, a further review of the data, using effect size as a measure, concluded that Oehen had been overly conservative and the results were indicative of MDMA psychotherapy providing substantial improvements for treatment-resistant PTSD (15).

Further teams in the USA, Israel and Canada then began conducting Phase 2 MDMA trials for PTSD. In 2018 a team based in Boulder, Colorado, USA submitted their results of a dose response model from multiple therapy teams on 28 participants (16). Two active doses (100 and 125 mg) were compared with a low dose (40 mg) session, and later the low dose group crossed over for three open-label active dose sessions. The active groups had the largest reduction in CAPS scores at the primary endpoint. The results at the primary endpoint were not significant, but at the 12 month follow-up the difference from baseline did reach significance. There were no drug-related serious adverse events and the treatment was well-tolerated. A further study demonstrated successful treatment of veterans and first responders with treatment-resistant PTSD (17). All of the contemporary MDMA-assisted psychotherapy studies to date have only been carried out on relatively small numbers of patients. Despite the consistently positive results and good tolerability of the treatments described in these studies above, larger, multisite trials are necessary to demonstrate the level of clinical efficacy and safety required to see MDMA become a licensed medicine. This phase of clinical MDMA research is now underway.

In collaboration with the Food and Drugs Administration (FDA) in America and the European Medicines Agency (EMA) in Europe, the pooled data from all of the MAPS-sponsored Phase 2 trials formed the basis for expansion into multi-site Phase 3 trials of MDMA therapy for PTSD, with FDA-granting Breakthrough Therapy designation. Study centers for the MAPS phase 3 programme in the USA are now underway. The European sites—in the UK, Netherlands, Germany and the Czech Republic—are in the process of seeking approvals and are projected to start later in 2019, putting MDMA on course to becoming a licensed treatment in 2021 (18).

## The Safety of Clinical MDMA

In the early 2000s—at the height of Ecstasy's demonization in the media—a debate around safety dominated the scientific and popular literature. But comparing clinical MDMA use with recreational ecstasy carries no scientific validity. Clinical subjects are screened, monitored throughout, are given pure drug and are closely followed-up for months afterwards. In contrast recreational ecstasy use frequently involves impure samples of MDMA, taking multiple other drugs and often paying little attention to the physiological aspects of the drug experience. Nevertheless, even when one *does* look at recreational ecstasy, which is used by around 750,000 people every weekend in the UK (19), the rates of morbidity and mortality are low. One study demonstrated that after removing confounding factors of concomitant drugs, there were only three deaths per year attributed solely to MDMA (20). Further studies that control for confounding factors show no evidence of neurotoxicity with MDMA when used in isolation (21) and no lasting neurocognitive impairments (22). Given that Ecstasy has such widespread use—second only to cannabis in popularity as an illicit drug—these epidemiological and experimental data demonstrate its relative safety.

Despite the absence of evidence for chronic adverse effects from clinical MDMA therapy, acutely the MDMA experience may be associated with transient neurocognitive effects, including verbal and spatial memory deficits, slow processing speeds and executive functioning impairments (23). But these resolve after the acute subjective psychological effects of the drug have worn off (24). Over 1,600 doses of clinical MDMA have being administered in research settings in recent years, with only one report of a drug-related self-limiting serious adverse event and no deaths (18). A large analysis on 166 subjects given MDMA in a controlled setting by Vizeli et al. (25), demonstrated no serious adverse events and showed that MDMA “produced predominantly acute positive subjective drug effects.” The analysis also showed that subjective negative drug effects and other adverse effects were significantly more common in women. The paper concluded that, “MDMA administration was overall safe in physically and psychiatrically healthy subjects and in a medical setting.”

Compared to other stimulants (particularly cocaine, amphetamine and methamphetamine) addiction to MDMA is very rare. And in the last 15 years of clinical studies with medical MDMA, illicit use of ecstasy after having used it clinically is seldom observed (13).

Clinical MDMA administration typically causes increased blood pressure and heart rate, increased body temperature (25–27), jaw tightness, bruxism, reduced appetite, poor concentration, and impaired balance (12). More serious adverse effects have not been observed in the last 15 years of monitored sessions with clinical MDMA (28). Similarly, the low mood, irritability and fatigue described by Ecstasy users (and dubbed the “mid-week blues”) is rarely observed in the clinical setting (13, 29), though low mood has also been reported in healthy subjects after administration of MDMA in controlled settings (30–32). Studies suggest these “blues” are related to recreational users missing sleep, dancing excessively, using other drugs (including alcohol), and going without food (33, 34)—none of which occur in a clinical setting.

## Mechanism of Action of Clinical MDMA

Multiple receptors, neurotransmitters and intermediary processes probably account for MDMA's effects. MDMA mainly acts as a releaser of serotonin (5-HT) and noradrenaline, and to a lesser extent also of dopamine (35, 36). Typical effects of MDMA can be predominantly attributed to the activation of the 5-HT system (31, 37, 38).

Activity at 5-HT_1A_ and 5-HT_1B_ receptors attenuates feelings of depression and anxiety, reduces the amygdala fear response and increases levels of self-confidence (39). Increased feelings of closeness, greater compassion and increased empathy for oneself and others further contribute to positive mood (40, 41). Increased dopamine and noradrenaline raise levels of arousal and awareness (42, 43), motivating engagement in therapy and promoting fear extinction (44).

MDMA's effects at alpha-2 receptors, which contribute to the drug's effects on thermoregulation (45), may also contribute a paradoxical relaxation/sedation effect (46), which could be beneficial in the context of trauma-induced hypervigilance. While adrenergic alpha-1 receptors are involved in the thermogenic response to MDMA in humans (41), alpha-2 receptors do not appear to be critically involved in the psychological effects of MDMA in humans (47).

MDMA has been shown to facilitate the release of oxytocin, the hormone associated with early infantile bonding, which may increase levels of empathy and closeness (48–52) and dampen fear-related amygdala activity, causing a decrease in stress response and social anxiety (53, 54).

Animal studies have demonstrated MDMA increases fear extinction through a mechanism dependent on elevated levels of brain derived neurotropic factor (BDNF) in the amygdala (55, 56), which might account for the observed phenomenon of MDMA psychotherapy allowing for patients' safe recall of painful emotional memories, that are usually avoided due to the overwhelming negative affect that usually accompanies recall of such events. Increased prosocial feelings (57), improved tolerance for unpleasant memories (58) and enhanced empathy and self-compassion (59), can promote a strong therapeutic alliance to effectively process traumatic memories.

In summary, the combined pharmacological effects of MDMA and the associated subjective psychological experience provide a unique selective impairment of the fear response whilst leaving the other faculties intact. Therefore, MDMA could be “the perfect drug for trauma-related psychotherapy” (60).

## How We Carry Out Mdma-Assisted Psychotherapy

Psychotherapy with MDMA borrows much of its methodology from the earliest research with LSD in the 1950s. The concept of set and setting is central to the totality of the user's experience; where set refers to the user's mindset and setting refers to the environment in which the drug is taken. Much effort goes into developing the optimum psycho-environmental conditions for a clinical MDMA-assisted session (61). A comprehensive study with a 125 mg MDMA dose taken by 166 subjects in a clinical environment, showed 64% of the subjects gave reports that the they found the controlled setting reassuring and it made them feel safe (25).

Therapeutic sessions with MDMA are typically delivered by a male-female co-therapist dyadic pair. However, a recently completed study of MDMA-assisted Psychotherapy combined with Cognitive Behavioral Combined Therapy for couples in which one person had PTSD, used some co-therapy teams with two female therapists (https://clinicaltrials.gov/ct2/ show/NCT02876172). The drug-assisted sessions are non-directive; encouraging the patient to go with the experience. The medicine seems to catalyze the patient's innate healing ability, which does the work (7, 62). The therapists create a sense of safety and communicate trust in their patient's ability to explore their issues. Eyeshades are frequently employed in MDMA-assisted sessions and the use of music played through headphones is commonplace. Physiological observations such as regular measurements of blood pressure and temperature are also commonplace throughout the MDMA experience.

As well as the MDMA-assisted sessions, the non-drug therapeutic sessions that make up a total course of MDMA psychotherapy are essential for preparation before taking the drug and subsequent integration of the emergent material after the drug sessions. Taken on its own, without adequate pre-drug preparation or post-drug support, MDMA is less likely to have a positive beneficial effect [Mithoefer M—Personal Communication: ‘Our observation in Phase 2 clinical trials is that the preparation and follow-up visits are often crucial because the nature of this therapeutic process is that symptoms can increase afterMDMAassisted sessions (as they can in any deep processing of trauma), and without proper support this could lead to deterioration and risk of suicide for a subset of people.With proper support these challenges are ultimately useful and part of the healing trajectory rather than an adverse outcome' (2018)]. In training MDMA Therapists for the future, MAPS are currently leading the way with their manualised approach for MDMA-assisted psychotherapy for PTSD.

## Future Directions: Broadening MDMA Beyond PTSD

Up till now most MDMA therapy research has been conducted with patients with PTSD. But many people suffering with other chronic mental disorders will describe some degree of pre-morbid trauma, often secondary to sexual or physical child abuse, or more commonly emotional abuse and neglect, which are no less damaging to a person's subsequent development (63). Given that such child maltreatment is particularly prevalent in cases of adult addictions (64), we are now exploring the potential role for MDMA therapy in cases of adult alcohol use disorder.

Alcohol use disorder represents a serious clinical, social and personal burden on its sufferers and a significant financial strain on society. Current treatments, both psychological and pharmacological, are poor, with high rates of relapse after medical detoxification and dedicated treatment programs. The earliest historical roots of psychedelic drug-assisted psychotherapy in the 1950s for alcoholism were associated with LSD-assisted psychotherapy (65). Indeed, Bill Wilson, the founder of *Alcoholics Anonymous*, testified to the powerful potential of psychedelic-assisted therapies for treating alcoholism (66). And contemporary pilot studies with psilocybin therapy for alcohol addiction (67) and psilocybin therapy for nicotine addiction (68) have demonstrated positive results. But MDMA-assisted psychotherapy has never been explored as a treatment for any form of substance use disorder. However, MDMA could be well suited to allow a patient using alcohol as a form of self-medication against a history of childhood trauma to explore and address painful memories without being overwhelmed by negative affect. Furthermore, the acute psychological effects of MDMA, which are typically less perceptually disturbing than those produced by classic psychedelics, may be more easily tolerated by some people. Given that compliance is a critical part of addiction therapy, there are good grounds for exploring MDMA therapy for alcoholism (69). However, it must be borne in mind that the cardiovascular tolerability of MDMA is lower compared with hallucinogens (25, 50), which prompts the requirement for more robust vital signs monitoring during MDMA therapy compared to classic psychedelic drug-assisted psychotherapy.

The capacity for MDMA to increase feelings of empathy and compassion for the self and others may contribute to improved self-awareness and subsequently reduce the denial of alcohol misuse (70). Similarly, MDMA has been shown to increase feelings of mindfulness, which has been increasingly explored as a potential approach for treating alcohol use disorder (71). This is the hypothesis behind the UK's first ever clinical MDMA Therapy study, the Bristol-Imperial MDMA-for-Alcoholism (BIMA) study (69). The BIMA study enrols participants into an 8-week course of supportive psychotherapy employing elements of Motivational Interviewing. As with all psychedelic-drug assisted psychotherapy courses, most of therapeutic sessions are face-to-face *non*-drug-assisted sessions. Only on two occasions participants are administered open-label sessions of MDMA-assisted therapy. On each drug-assisted session participants receive an initial dose of 125 mg MDMA, followed 2 h later by a “booster dose” of 62.5 mg to prolong the experience. Throughout the drug-assisted session, vital signs, including blood pressure and body temperature, are monitored. Participants remain in the treatment center overnight after taking MDMA. Mood, sleep and suicide risk are monitored daily for a week. Participants are followed-up for 9-months post-detox, and outcome measures include safety and tolerability data, quality of life measures, physical and mental health status and drinking behaviors (69). This study will be completed by the end of 2019.

Another area of contemporary research with MDMA therapy has explored the potential for relief of social anxiety associated with autism, in a MAPS-sponsored randomized, double-blind, placebo-controlled pilot study completed at Harbor-UCLA Medical Center and Stanford University (72). One of the cardinal features of autism is a tendency for a sufferer to lack empathy. It is a recognized anecdotal observation that autistic adults often report reduced empathy-impairments during, and for some time after, taking MDMA (72).

Other contemporary areas of research with MDMA therapy include the potential for MDMA-assisted psychotherapy in treating mood disorders (73) and, relatedly, as an alternative to electro-convulsive therapy (74).

## Summary and Conclusion

As MAPS pushes ahead with Phase 3 studies in the USA and Europe for MDMA therapy for PTSD, we are seeing a broadening of the clinical possibilities for the compound. Meanwhile psychiatrists are increasingly recognizing the role played by early psychological trauma in a range of mental disorders beyond that of PTSD (69).

Due to its association with recreational Ecstasy, MDMA has a long-standing label of controversy in the UK. But this narrative must be tackled; partly because the compound is demonstrably safe and efficacious in the clinical setting and partly because politics and erroneous media-driven opinion must not be allowed to dictate the progress of medical research (75). Like everything else, MDMA is not 100% safe. As with all medical interventions—from sticking plasters to cancer chemotherapy—MDMA may be simultaneously both invasive and beneficial and therefore the same principles of evidence-based clinical governance must be applied to psychedelics as they are to other therapeutic approaches (76). Clinical MDMA and recreational ecstasy are incomparable in terms of drug purity, administration and the screening and monitoring of selected participants. “Prohibition of MDMA and other illicit drugs increases, not reduces, the potential harms of recreational drug use (75), adds unnecessary costs that put research beyond the financial capabilities of many academic institutions, and therefore hold back progress (77).”

There remains much work to be done to convince critics that a compound that is experienced recreationally by so many people may also, in its clinical form, have benefits for patients suffering with treatment-resistant mental disorders. Meanwhile, psychedelic culture is enjoying a palpable renaissance in both medicine and the media. Against this backdrop, psychiatry and society continue to be burdened with far-from-perfect treatment outcomes for many mental disorders. In this context, given the clinical burden, the lack of treatment efficacy and their continued distress, perhaps the only question we should be asking is: Can we afford *not* to explore MDMA therapy for our worthy patients?

## Author Contributions

BS wrote the main body of the paper, including citations. LH and DN added further commentary and reviewed and edited the paper.

### Conflict of Interest Statement

The authors declare that the research was conducted in the absence of any commercial or financial relationships that could be construed as a potential conflict of interest.
